# Sero-reactivity to three distinct regions within the hepatitis C virus alternative reading frame protein (ARFP/core+1) in patients with chronic HCV genotype-3 infection

**DOI:** 10.1099/jgv.0.001727

**Published:** 2022-03-01

**Authors:** Mosaab E. A. Elsheikh, C. Patrick McClure, Alexander W. Tarr, William L. Irving

**Affiliations:** ^1^​ School of Life Sciences, Faculty of Medicine and Health Sciences, The University of Nottingham, Nottingham, UK; ^2^​ Wolfson Centre for Global Virus Infections, The University of Nottingham, Nottingham, UK; ^3^​ NIHR Nottingham Biomedical Research Centre, Nottingham University Hospitals NHS Trust and the University of Nottingham, Nottingham, UK

**Keywords:** hepatitis C, alternative reading frame protein, HCV genotype 3, disease progresssion

## Abstract

Hepatitis C virus (HCV) infection affects more than 71 million people worldwide. The disease slowly progresses to chronic, long-term liver injury which leads to hepatocellular carcinoma (HCC) in 5 % of infections. The alternative reading frame protein (ARFP/core+1) is encoded by a sequence overlapping the HCV core gene in the +1 reading frame. Its role in hepatitis C pathogenesis and the viral life cycle is unclear, although some observers have related its production to disease progression and the development of HCC. The aim of this study was to determine whether ARFP is immunogenic in patients with chronic HCV genotype 3 infection and to assess whether sero-reactivity is associated with disease progression, particularly to HCC. Immunogenic epitopes within the protein were predicted by a bioinformatics tool, and three −20 aa length-peptides (ARFP-P1, ARFP-P2 and ARFP-P3) were synthesized and used in an avidin-biotin ARFP/core+1 peptide ELISA. Serum samples from 50 patients with chronic HCV genotype 3 infection, 50 genotype-1 patients, 50 HBV patients and 110 healthy controls were tested. Sero-reactivity to the ARFP peptides was also tested and compared in 114 chronic HCV genotype-3 patients subdivided on the basis of disease severity into non-cirrhotic, cirrhotic and HCC groups. Chronic HCV genotype-3 patients showed noticeable rates of reactivity to ARFP and core peptides. Seropositivity rates were 58% for ARFP-P1, 47 % for ARFP-P2, 5.9 % for ARFP-P3 and 100 % for C22 peptides. There was no significant difference between these seroreactivities between HCV genotype-3 patients with HCC, and HCV genotype-3 patients with and without liver cirrhosis. Patients with chronic HCV genotype-3 infection frequently produce antibodies against ARFP/core+1 protein. ARFP peptide reactivity was not associated with disease severity in patients with HCV genotype-3. These results support the conclusion that ARFP/core+1 is produced during HCV infection, but they do not confirm that antibodies to ARFP can indicate HCV disease progression.

## Background

Hepatitis C virus (HCV) is an enveloped, ssRNA virus, which belongs to the family *Flaviviridae*, within the genus hepacivirus [1]. Eight genotypes and more than 70 subtypes have been described [2–5]. Chronic HCV infection is a leading cause of hepatocellular carcinoma (HCC), end-stage liver disease and liver-related mortalities [6, 7]. HCV genotype-3, in comparison with other genotypes, is associated with an increased risk of liver steatosis, cirrhosis and HCC [8–10]. HCV-related pathogenesis of HCC is understood to be a long term, multi-step process involving chronic infection, liver fibrosis and cirrhosis followed by malignant transformation [11–14]. Several viral proteins have been shown to have potential roles in the pathogenesis of HCC. HCV core protein was mostly implicated in induction of malignant transformation due to its interaction with many intracellular molecules that are involved in oncogenesis [15–19]. In addition, core protein is also thought to be involved in the development of liver steatosis as well as insulin resistance [20, 21]. The HCV genome encodes an alternative reading frame protein (ARFP) that overlaps the core gene on the +1 reading frame. Previous studies showed that this sequence may be expressed during the natural course of HCV infection and may evoke explicit, specific immune responses [22–30]. The biological role of the ARFP/core+1 protein remains unknown. However, due to the fact that it is derived from an overlapping core sequence, it was postulated that ARFP/core+1 may share certain biological functions linked to the core protein, including hepato-carcinogenesis [30–33].

The expression of ARFP/core+1 throughout the course of HCV infection has been studied indirectly by observing the generation of specific humoral and cellular mediated immune responses in HCV patients [22–29]. However, few studies have related ARFP/core+1 expression clinically to HCC. Dalagiorgou *et al*. observed the prevalence of high-titre anti-ARFP/core+1 antibodies in patients with HCC in comparison to HCV-infected patients without HCC. The authors proposed that ARFP may therefore have a potential role in the pathogenesis of HCC [34, 35]. Nevertheless, the antibody prevalence of ARFP/core+1 in patients with HCV genotype-3 HCC has not been extensively reported.

One approach for assessing seroreactivity is via the use of custom-made linear peptides, spanning the potential immunogenic regions of the ARFP/core+1 protein [24]. This approach produced more specific signals [24] but with lower reactivity compared with recombinant antigens, as reported by Walewski *et al.* who used ARFP/core+1 synthetic HCV-1a peptides in an immunoblot assay [28], Morice *et al.* who used a genotype-1b synthetic peptide composed of 99 aa, in an ELISA, [25] Chuang *et al.* who used an HCV-2 14 aa peptide [23] and Qureshi *et al.* who used an HCV-3 20 aa synthetic peptide [36].

This study aimed to assess the humoral immune response to ARFP/core+1 in a large cohort of genotype-3 HCV-infected patients with defined clinical outcomes to see whether the detection of such antibodies changes over the disease course.

## Methods

### Synthesis of ARFP/core+1 peptides via prediction of B-cell epitopes

ARFP/core+1 genotype-3 sequences obtained from GenBank were entered into Protean 3D application (DNASTAR Lasergene) to predict potential antigenic sites within the ARFP/core+1 consensus amino acid sequence based on formulations described by Jameson and Wolf [37] (Fig. S1, available in the online version of this article). Three peptides of length 20 aaa were then selected: ARFP-1 (positions 5–24), ARFP-2 (positions 56–75) and ARFP-3 (positions 121–140). The HCV recombinant protein C22-3 is encoded by the core region of the HCV genome [38–40]. A core peptide was also synthesized spanning positions 6–25, KPQRKTKRNTIRRPQDVKFP derived from the commercial sequence of the known antigenic C22-3 peptide (amino acid sequence 2–120) as a positive control. All peptide sequences are shown in Table 1. Peptide synthesis and purification was carried out by Mimitopes. The peptide stocks were prepared at a concentration of 10 mg ml^−1^ in DMSO and stored at −25 °C.

**Table 1.** Sequences of peptides

Site of peptide	Peptide sequence
ARFP-1=positions 5–24	NLKEKPKETPSVAHRTSSSR
ARFP-2=positions 56–75	SLADDDSLSPRRVGAKAGPG
ARFP-3=positions 121–140	SSIPLRADSPTSWGTSRSSA
Core=positions 6–25	KPQRKTKRNTIRRPQDVKFP

### Patients and samples

Serum samples from HCV-infected patients were provided by the HCV Research UK biobank [41] and from the Microbiology Department, Queen’s Medical Centre, Nottingham University Hospital NHS Trust. A number of different subgroups of sera were tested, distributed as follows:

Sera used for assay validity:

50 chronic HCV genotype-3-positive patients

50 chronic HCV genotype-1-positive patients

50 HBV DNA-positive HCV-negative patients

50 HCV-negative blood donors.

Sera used for comparing ARFP sero-reactivity according to disease stage:

HCV-infected HCC patients, *n*=51

HCV-infected patients with cirrhosis but no HCC, *n*=39

HCV-infected patients without cirrhosis or HCC, *n*=24

110 HCV-negative blood donors.

### NeutrAvidin-biotin ARFP peptide ELISA assay

In total, 50 µl of 5 µg NeutrAvidin ml^−1^ in carbonate/bicarbonate buffer was added to 96-well Maxisorp ELISA plates (Nunc) and incubated overnight at 4 °C. The plates were blocked with 200 µl of 3 % BSA in 0.05 % Tween20 in PBS (PBS/T) for 2 h at room temperature. The plates were washed once with 200 µl PBST. Then 50 µl of the diluted biotinylated ARFP-1, -2 and -3, and C22 peptides at a concentration of 1 µg ml^−1^ was added and incubated for 1 h at room temperature. The plate was washed three times with 200 µl PBST. Then 50 µl of the test sera at a dilution of 1:100 was added and incubated at room temperature for 1 h. The plate was washed three times with 200 µl PBST. Then 50 µl of goat anti-human IgG conjugated to alkaline phosphatase (Sigma) was added at a dilution of 1 : 2000 in the blocking buffer for 1 h at room temperature. The plate was washed three times with 200 µl PBST. The binding was visualized with the addition of 50 µl *p*-nitrophenyl phosphate substrate (pNPP) (Sigma) and incubation in a dark place for 20 min at room temperature. The absorbance reflected by the optical density (OD) was measured at 405 nm with an Omega FLUOROSTAR plate reader.

### Statistical analysis

The mean, median and standard deviation for the HCV-negative signals were calculated using GraphPad Prism. For each ELISA experiment, the cut-off value was determined as being the mean value of the group of HCV-negative control sera plus 2 sd. The Kruskal–Wallis test for non-parametric data using multiple comparisons was used to compare the reactivity data in different groups. This is adjusted for repeated measures using Dunn’s multiple comparison test. *P* values <0.05 were considered statistically significant.

## Results

### Identification of B-cell epitopes within ARFP

The predicted immunogenic sites within the genotype 3 consensus sequence of ARFP were located mostly within amino acids at positions 5–35, 55–80, 90–95, 110–115 and 120–142. These are shown in Fig. S1, together with the location of the three peptides chosen for use in this study (5–24, 56–75 and 121–140). The amino acid sequences are shown in Table 1.

### HCV patients produce antibodies to ARFP

**Fig. 1.**
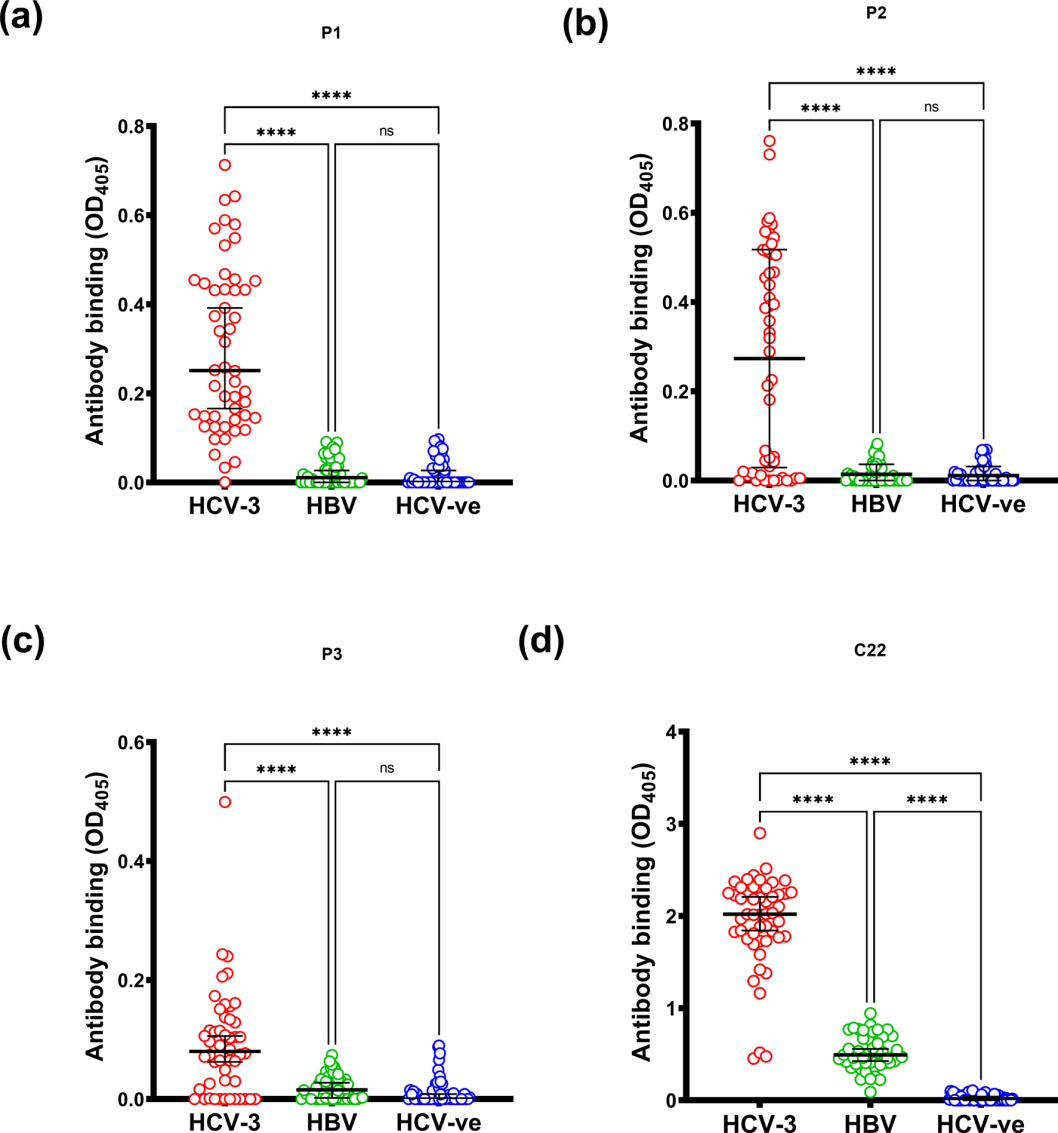
Seroreactivities of the ARFP and core peptides in three study groups, 50 HCV genotype 3-positive sera, 50 HBV-positive sera and 50 healthy control sera. The figures quoted above each set of results are the percentage of samples deemed to be seropositive. The cut-off was set as the mean of HCV-negative sera plus 2 sd (above OD 0.125). The level of reactivity of the three groups was compared using the Kruskal–Wallis test for non-parametric data adjusted for repeat measures with Dunn’s multiple comparison test. *P<0.05; **P<0.01; ***P<0.001; ****P<0.0001.

The reactivity of sera from patients with chronic HCV genotype 3 infection, chronic HBV infection and blood donors (negative for both HCV and HBV infection) against the core and each of the three ARFP peptides is shown in Fig. 1. The sera from HCV patients showed significantly increased reactivity to all four peptides as compared to the HBV and control sera. Reactivity against core was highest, followed by that against ARFP1, ARFP2 and ARFP3.

### Sero-positivity rates are dependent on genotype when using genotype-3-derived peptides

**Fig. 2.**
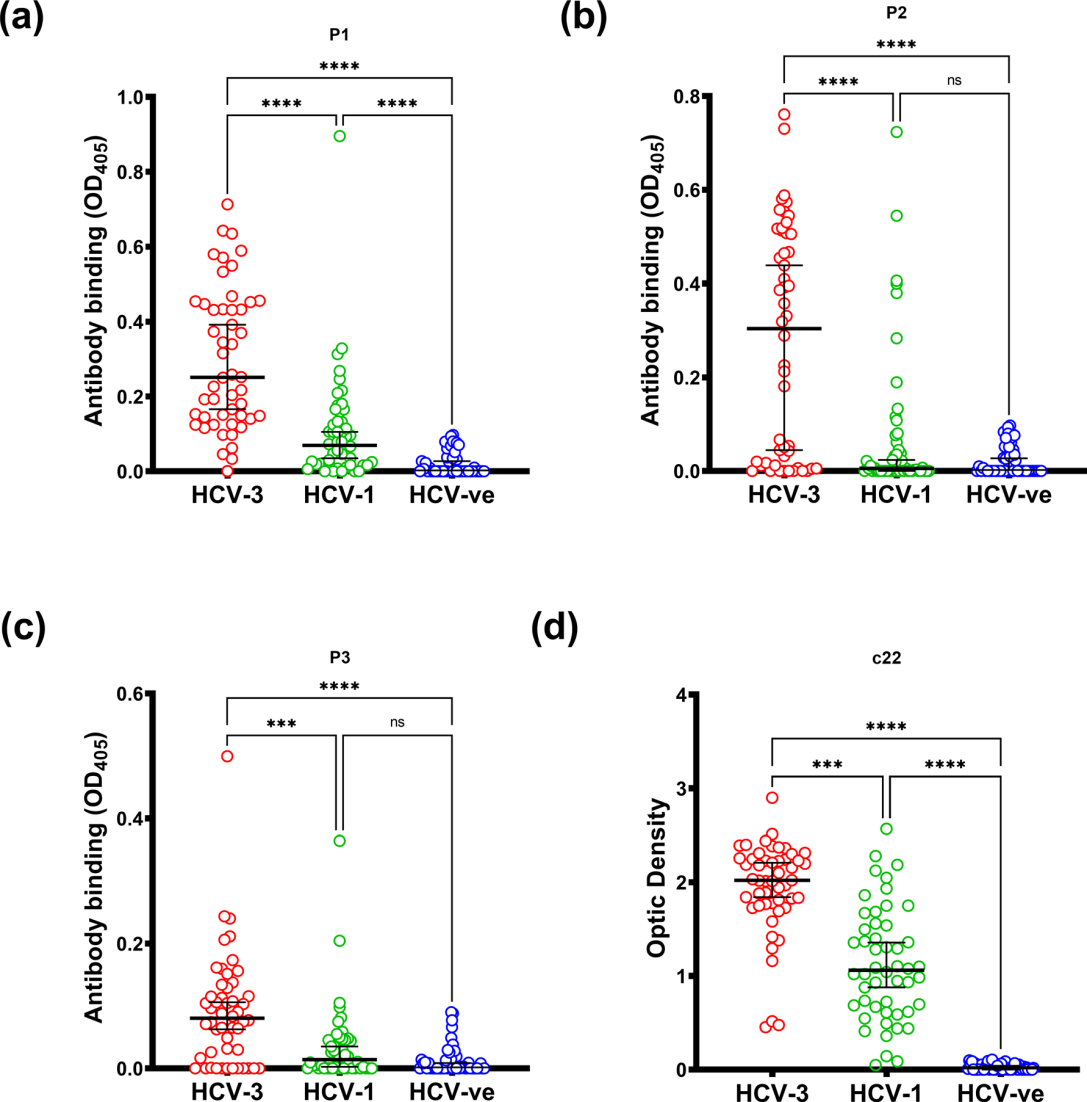
The seroreactivity of ARFP and core peptides in three study groups, 50 HCV genotype 3 sera, 50 HCV genotype 1 sera and 50 healthy control sera. The ARFP peptides were compared with corresponding amino acid sequences from genotype 1 consensus sequences within the +1 reading frame. The figures quoted above each set of results are the percentage of samples deemed to be seropositive. The cut-off was set as the mean of HCV-negative sera plus 2 sd (above OD 0.125). The level of reactivity of the three groups was compared using the Kruskal–Wallis test for non-parametric data adjusted for repeat measures with Dunn’s multiple comparison test. *P<0.05; **P<0.01; ***P<0.001; ****P<0.0001.

The reactivity of samples to the ARFP peptides from patients infected with genotype-1 was significantly lower compared to genotype-3 sera (*P*<0.00001, Fig. 2). In total, 96 % of the genotype 1 sera were reactive with the C22-derived peptide. The consensus genotype 3 ARFP-P1 sequence varied from its corresponding genotype-1 sequence by 6 aa, ARFP-P2 by 9 aa and ARFP-P3 by 3 aa (Fig. 2).

### Comparing genotype-3 patients at different disease stages revealed no difference in sero-reactivity or seroprevalence rates

**Fig. 3.**
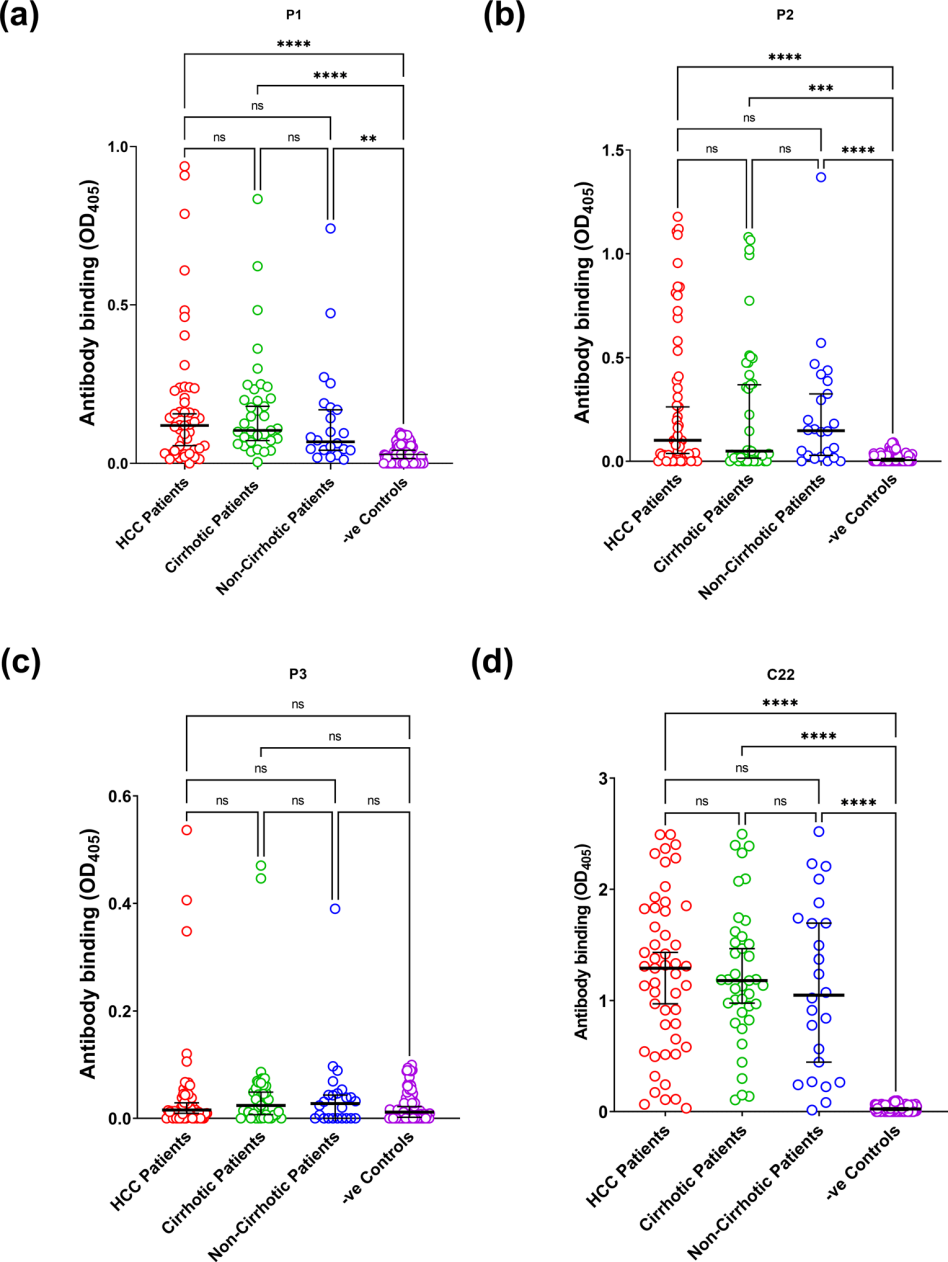
The peptide sero-reactivity in patients with chronic HCV genotype 3 infection. The signal cut-off was determined by calculating the mean of the negative control sera plus 2 sd. Comparison of the seroreactivity between HCC, cirrhotic and non-cirrhotic patients was carried out using the Kruskal–Wallis test for non-parametric data adjusted for repeat measures with Dunn’s multiple comparison test. *P<0.05; **P<0.01; ***P<0.001; ****P<0.0001.

We tested samples derived only from patients infected with HCV genotype 3, but at differing disease stage (chronic hepatitis, cirrhosis, HCC). Patients with chronic hepatitis or cirrhosis were age- and sex-matched to those with HCC (Table 2). Reactivity to ARFP-peptide-1 was seen in 27 of 51 (52.9 %) sera from patients with HCC, 19 of 39 (48.7 %) from patients with cirrhosis, and eight of 24 (33.3 %) from the non-cirrhotic patients. Reactivity to ARFP-peptide-2 was seen in 24 (47 %), 17 (43.6 %) and 13 (54.1 %) sera respectively, and reactivity to ARFP-peptide-3 revealed the lowest reactivity of all, with sero-positive rates of 5.9, 5.1 and 4.2% respectively. There were no significant differences in the sero-prevalence rates to any of the three ARFP peptides between the HCC patients and the other groups. Reactivity to the core C22 was seen in 49 (96 %) of the HCC patients, all of the cirrhotic patients and 22 (96 %) of the non-cirrhotic patients (Table 3, Fig. 3). Finally, we combined the patients in the cirrhotic group with those in the HCC group (because HCC associated with HCV infection almost always occurs in the presence of cirrhosis) and compared reactivity in this combined cirrhosis cohort with that in the chronic hepatitis cohort. There was no significant difference in reactivity for any of the four peptides (data not shown).

**Table 2.** Age and gender distribution of the chronic HCV genotype 3 cohort

		HCC patients (*n*=51)	Cirrhotic patients (*n*=39)	Non-cirrhotic patients (*n*=24)
Gender	Female	18 (35.3 %)	9 (23.1 %)	9 (37.5 %)
	Male	33 (64.7 %)	30 (76.9 %)	15 (62.5 %)
Age, years	Mean±sd	55±5.7	55±6.2	52±7.4
	31–40	0	0	2 (5.1 %)
	41–50	12 (23.5 %)	11 (28.2 %)	7 (8.3 %)
	51–60	34 (66.7 %)	24 (61.5 %)	11 (45.8 %)
	61–70	5 (9.8 %)	2 (5.1 %)	4 (16.6 %)
	71–80	0	2 (5.1 %)	0

**Table 3.** The prevalence of antibodies against ARFP and core peptides among chronic HCV genotype 3 patients

	HCC patients (*n*=51)	Cirrhotic patients (*n*=39)	Non-cirrhotic patients (*n*=24)
ARFP-P1	52.9%	48.7%	33.3%
ARFP-P2	47%	43.6%	54.1%
ARFP-P3	5.9%	5.1%	4.2%
Core-C22	96%	100%	96%

## Discussion

After more than a decade since its discovery, there are many controversies regarding production of the ARFP/core+1 protein during HCV infection and its role in relation to the pathogenesis of disease [30]. Several independent studies showed that chronic HCV patients can elicit specific humoral and cell-mediated immune responses toward ARFP/core+1, indirect evidence for its expression during HCV infection [22–28]. Similarly, in this study, we have demonstrated that patients infected with HCV generate a humoral immune response to ARFP/core+1, indicating that the protein must be expressed during chronic infection. None of the HBV-infected or blood donor samples reacted with any of the three ARFP peptides (Fig. 1). Overall, HCV sera showed less reactivity against ARFP/core+1 peptides compared to the core-derived peptide. Our data also indicate the importance of using genotype-specific ARFP/core+1 linear antigens when assessing sero-reactivity. Our genotype-3-derived ARFP peptides 1, 2 and 3 differ from their consensus genotype-1 counterparts by 6, 9 and 3 aa respectively. Reactivity of the genotype 1 sera with genotype-3-derived ARFP peptides was significantly less than that of the genotype 3 sera (Fig. 2). In contrast, there was notable reactivity of genotype 1 and 3 sera against the core-derived peptide, probably due to the higher degree of sequence conservation among different HCV subtypes.

There are alternative ways of identifying immunogenic regions within proteins other than the Protean 3D application used in this report, and other algorithms may have identified different antigenic regions within ARFP. However, the peptides we derived from our approach clearly generated an immune response in HCV-genotype 3-infected patients. Peptide ARFP-P3 was the least antigenic. One possible explanation for this is that perhaps our patients were infected with HCV sequences in which the ARFP ORF contained a stop codon prior to the end of ARFP-P3. To address this, we searched the Los Alamos HCV sequence database (https://hcv.lanl.gov/content/index) for genotype 3 Core/ARFP nucleotide sequences that were deposited from the UK or Europe. Sequences were translated in the ARFP reading frame and the occurrence of stop codons was determined. There were 61 samples with the relevant sequence data available. Of these, 25 were from the UK, of which four contained a premature stop codon. Thus, this is unlikely to explain the low reactivity to ARFP-P3 in our cohort.

Clinically, there was no evidence that the antibody response to ARFP/core+1 changes significantly in relation to disease progression, which is in contrast to previously published data. Seroprevalence to ARFP/core+1 will be influenced by the duration of infection and whether the infecting HCV genotype is the same or different from the ARFP/core+1 antigen used in the assay to detect antibodies. All of our cohort of clinically well-characterized patients had genotype 3 infection, corresponding to the origin of our peptides; the different clinical groups (chronic hepatitis, cirrhosis, HCC) were well age-matched (Table 2), in contrast to other reports [22–24, 28, 36]. Kassela *et al.* [42] reported higher seroprevalence rates in advanced versus mild cirrhosis, but it is not clear whether all cirrhotic patients were infected with the same genotype.

These results are supported by observations by Qureshi *et al.* who tested sera from 88 HCV genotype-3 patients using 20 recombinant ARFP overlapping peptides – of 20 aa long – spanning the whole of the ARFP sequence. They revealed that the region 60–80 (similar to our ARFP-P2 56–75) was the most immunogenic, compared with the region 1–20 (similar to our ARFP-P1 5–24) and region 120–140 (similar to our ARFP-P3 121–140) [34]. However, there was a difference in reactivity between their 120–140 region, which was more immunogenic than our ARFP-P3 and ARFP-P1.

A different observation was reported by Dalagiorgou *et al.*, when they assessed the prevalence of anti-ARFP/core+1 in patients with HCC, compared with chronic, non-HCC HCV Greek patients. First, they created two recombinant, full-length, ARFP/core+1 proteins for genotypes 1a and 1b, in addition to the short form of ARFP/core+1 spanning 59 aa starting from position 85. They reported >50 % of 45 HCC patients were seropositive in comparison to 26 % of the 47 non-HCC patients, a significant difference between the two groups [34]. However, due to the wide disease spectrum of the non-HCC group studied, it was not clear in this study which chronic stage was associated with lower anti-ARFP/core+1 prevalence compared with the HCC group. In addition, no other serological control – for instance core – was used to compare the reactivity with ARFP/core+1. Most importantly, the two groups were not matched with regard to age (mean age of the HCC group was 65 years; that of the non-HCC group was 40 years), gender or HCV genotype, casting further concerns about the generalizability of their results.

All of our HCV-infected patients showed a high seroprevalence of anti-core antibodies, significantly greater than that against the three ARFP/core+1 peptides, suggesting that ARFP/core+1 expression occurs at lower rates compared with the core expression. Vassilaki *et al.* reported that various biological factors may control the expression of ARFP, including its sensitivity to intracellular proteasome degradation and downregulation by core, suggestive that ARFP may be produced in situations inauspicious for intracellular HCV replication [30]. It is known that HCC advances in individuals with chronic HCV genotype-3 infection after a mean of 20–30 years. As seropositivity rates to ARFP/core+1 did not vary significantly between age-matched genotype 3 patients with no cirrhosis, cirrhosis or HCC, this suggests that ARFP expression cannot be considered as a surrogate marker for HCV disease progression. However, more observations with larger cohorts are required to substantiate these results and to observe the T-cell responses as well.

In conclusion, HCV genotype-3 patients exhibited high rates of sero-prevalence to ARFP/core+1 and core peptides. There was no significant difference between these sero-reactivities in HCC and non-HCC groups. These results support that ARFP/core+1 is produced during HCV infection, but do not confirm that anti-ARFP/core+1 reactivity can be indicative for HCV disease progression or a marker for HCC development.

## Supplementary Data

Supplementary material 1

## References

[1] de Moura MC (1990). Non-A, non-B hepatitis: hepatitis C. Acta Med Port.

[2] Kato N (2000). Genome of human hepatitis C virus (HCV): gene organization, sequence diversity, and variation. Microb Comp Genomics.

[3] Smith DB, Bukh J, Kuiken C, Muerhoff AS, Rice CM (2014). Expanded classification of hepatitis C virus into 7 genotypes and 67 subtypes: updated criteria and genotype assignment web resource. Hepatology.

[4] Borgia SM, Hedskog C, Parhy B, Hyland RH, Stamm LM (2018). Identification of a Novel Hepatitis C Virus Genotype From Punjab, India: Expanding Classification of Hepatitis C Virus Into 8 Genotypes. J Infect Dis.

[5] Hedskog C, Parhy B, Chang S, Zeuzem S, Moreno C (2019). Open Forum Infectious Diseases.

[6] El-Serag HB, Kramer J, Duan Z, Kanwal F (2016). Epidemiology and outcomes of hepatitis C infection in elderly US Veterans. J Viral Hepat.

[7] El-Serag HB (2002). Hepatocellular carcinoma: an epidemiologic view. J Clin Gastroenterol.

[8] Torres HA (2012). Telaprevir containing regimen against hepatitis c virus infection in patients with hepatocellular carcinoma awaiting liver transplantation - a case series. Hepatology.

[9] Kanwal F, Kramer JR, Ilyas J, Duan Z, El-Serag HB (2014). HCV genotype 3 is associated with an increased risk of cirrhosis and hepatocellular cancer in a national sample of U.S. Veterans with HCV. Hepatology.

[10] Nkontchou G, Ziol M, Aout M, Lhabadie M, Baazia Y (2011). HCV genotype 3 is associated with a higher hepatocellular carcinoma incidence in patients with ongoing viral C cirrhosis. J Viral Hepat.

[11] El-Serag HB (2012). Epidemiology of viral hepatitis and hepatocellular carcinoma. Gastroenterology.

[12] Ramadori G, Saile B (2004). Portal tract fibrogenesis in the liver. Lab Invest.

[13] Burke KP, Cox AL (2010). Hepatitis C virus evasion of adaptive immune responses: a model for viral persistence. Immunol Res.

[14] Koike K (2005). Molecular basis of hepatitis C virus-associated hepatocarcinogenesis: lessons from animal model studies. Clin Gastroenterol Hepatol.

[15] Liu J, Ding X, Tang J, Cao Y, Hu P (2011). Enhancement of canonical Wnt/β-catenin signaling activity by HCV core protein promotes cell growth of hepatocellular carcinoma cells. PLoS One.

[16] Koike K (2007). Hepatitis C virus contributes to hepatocarcinogenesis by modulating metabolic and intracellular signaling pathways. J Gastroenterol Hepatol.

[17] El-Shamy A, Pendleton M, Eng FJ, Doyle EH, Bashir A (2016). Impact of HCV core gene quasispecies on hepatocellular carcinoma risk among HALT-C trial patients. Sci Rep.

[18] Joo M, Hahn YS, Kwon M, Sadikot RT, Blackwell TS (2005). Hepatitis C virus core protein suppresses NF-kappaB activation and cyclooxygenase-2 expression by direct interaction with IkappaB kinase beta. J Virol.

[19] Ray RB, Lagging LM, Meyer K, Steele R, Ray R (1995). Transcriptional regulation of cellular and viral promoters by the hepatitis C virus core protein. Virus Res.

[20] Moriya K, Fujie H, Shintani Y, Yotsuyanagi H, Tsutsumi T (1998). The core protein of hepatitis C virus induces hepatocellular carcinoma in transgenic mice. Nat Med.

[21] Perlemuter G, Sabile A, Letteron P, Vona G, Topilco A (2002). Hepatitis C virus core protein inhibits microsomal triglyceride transfer protein activity and very low density lipoprotein secretion: a model of viral-related steatosis. FASEB J.

[22] Bain C, Parroche P, Lavergne JP, Duverger B, Vieux C (2004). Memory T-cell-mediated immune responses specific to an alternative core protein in hepatitis C virus infection. J Virol.

[23] Chuang WCM, Allain JP (2008). Differential reactivity of putative genotype 2 hepatitis C virus F protein between chronic and recovered infections. J Gen Virol.

[24] Komurian-Pradel F, Rajoharison A, Berland J-L, Khouri V, Perret M (2004). Antigenic relevance of F protein in chronic hepatitis C virus infection. Hepatology.

[25] Morice Y, Ratinier M, Miladi A, Chevaliez S, Germanidis G (2009). Seroconversion to hepatitis C virus alternate reading frame protein during acute infection. Hepatology.

[26] Varaklioti A, Vassilaki N, Georgopoulou U, Mavromara P (2002). Alternate translation occurs within the core coding region of the hepatitis C viral genome. J Biol Chem.

[27] Troesch M, Jalbert E, Canobio S, Boulassel MR, Routy J-P (2005). Characterization of humoral and cell-mediated immune responses directed against hepatitis C virus F protein in subjects co-infected with hepatitis C virus and HIV-1. AIDS.

[28] Walewski JL, Keller TR, Stump DD, Branch AD (2001). Evidence for a new hepatitis C virus antigen encoded in an overlapping reading frame. RNA.

[29] Xu Z, Choi J, Yen TS, Lu W, Strohecker A (2001). Synthesis of a novel hepatitis C virus protein by ribosomal frameshift. EMBO J.

[30] Vassilaki N, Mavromara P (2009). The HCV ARFP/F/core+1 protein: production and functional analysis of an unconventional viral product. IUBMB Life.

[31] Hu W-T, Li H-C, Lee S-K, Ma H-C, Yang C-H (2013). Both core and F proteins of hepatitis C virus could enhance cell proliferation in transgenic mice. Biochem Biophys Res Commun.

[32] Sobesky R (2004). Study of intra-hepatic hepatitis c virus (HCV) core gene variability in hepatocellular carcinoma using laser capture microdissection. Hepatology.

[33] Alam SS, Nakamura T, Naganuma A, Nozaki A, Nouso K (2002). Hepatitis C virus quasispecies in cancerous and noncancerous hepatic lesions: the core protein-encoding region. Acta Med Okayama.

[34] Dalagiorgou G, Vassilaki N, Foka P, Boumlic A, Kakkanas A (2011). High levels of HCV core+1 antibodies in HCV patients with hepatocellular carcinoma. J Gen Virol.

[35] Dalagiorgou G (2008). Detection of humoral responses to the hepatitis c virus core+1 protein in patients with hcv-associated hepatocellular carcinoma. FEBS J.

[36] Qureshi H, Qazi R, Hamid S, Qureshi SA (2011). Identification of immunogenic regions within the alternative reading frame protein of hepatitis C virus (genotype 3). Eur J Clin Microbiol Infect Dis.

[37] Jameson BA, Wolf H (1988). The antigenic index: a novel algorithm for predicting antigenic determinants. Comput Appl Biosci.

[38] Pujol FH, Khudyakov YE, Devesa M, León G, Blitz-Dorfman L (1996). Characterization of the antibody reactivity to synthetic peptides from different parts of the hepatitis C virus genome. Viral Immunol.

[39] Van der Poel CL, Cuypers HT, Reesink HW, Weiner AJ, Quan S (1991). Confirmation of hepatitis C virus infection by new four-antigen recombinant immunoblot assay. Lancet.

[40] Hosein B, Fang CT, Popovsky MA, Ye J, Zhang M (1991). Improved serodiagnosis of hepatitis C virus infection with synthetic peptide antigen from capsid protein. Proc Natl Acad Sci U S A.

[41] McLauchlan J, Innes H, Dillon JF, Foster G, Holtham E (2017). Cohort Profile: The Hepatitis C Virus (HCV) Research UK Clinical Database and Biobank. Int J Epidemiol.

[42] Kassela K, Karakasiliotis I, Charpantidis S, Koskinas J, Mylopoulou T (2017). High prevalence of antibodies to core+1/ARF protein in HCV-infected patients with advanced cirrhosis. J Gen Virol.

