# Supplementary material for: Sero-reactivity to three distinct regions within the hepatitis C virus alternative reading frame protein (ARFP/core+1) in patients with chronic HCV genotype-3 infection
Source: J Gen Virol. 2022 Mar 1;103(3):001727. doi: 10.1099/jgv.0.001727 (PMC9176264; doi:10.1099/jgv.0.001727)

Supplementary Figure 1: DNA STAR ™ Protean algorithm prediction of potential antigenic sites within the genotype 3 ARFP/core + 1 primary amino acid consensus sequence (1A), based on formulations described by Jameson and Wolf. The values on the Y axis represent the "Antigenic Index", used to generate a linear surface contour profile of the protein (1B). The ARFP/core + 1 antigenic index takes account of the impact of parameters including entropy plots determined by deduced amino acid sequences (1C) hydrophilicity (1D), surface probability, backbone flexibility and secondary structure [41]

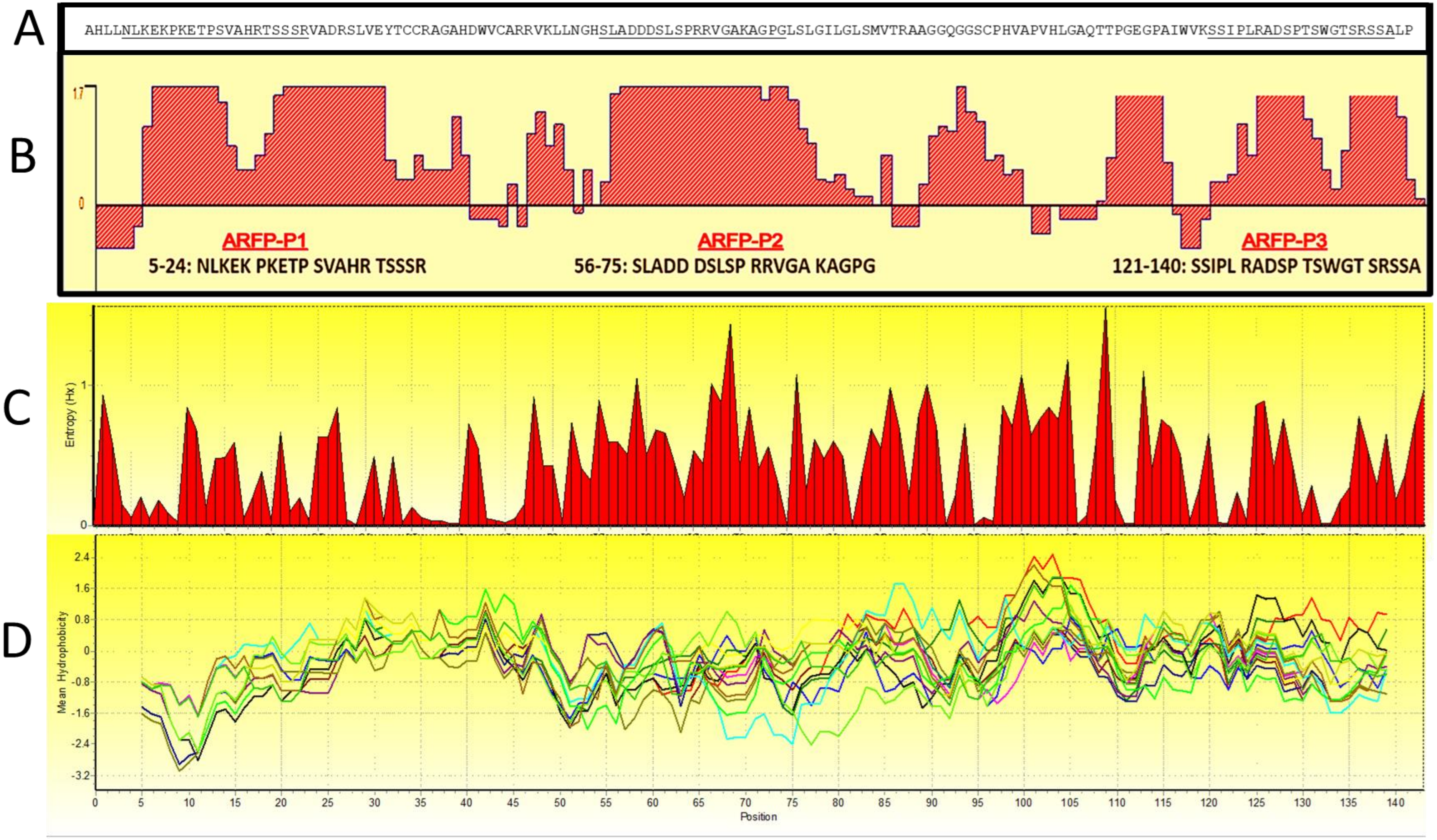

Supplement: Supplementary material 1 [file jgv-103-1727-s001.pdf]
